# Genome-wide identification and in-silico expression analysis of carotenoid cleavage oxygenases gene family in *Oryza sativa* (rice) in response to abiotic stress

**DOI:** 10.3389/fpls.2023.1269995

**Published:** 2023-10-25

**Authors:** Muhammad Zeshan Haider, Adnan Sami, Muhammad Shafiq, Waheed Anwar, Sajid Ali, Qurban Ali, Sher Muhammad, Irfan Manzoor, Muhammad Adnan Shahid, Daoud Ali, Saud Alarifi

**Affiliations:** ^1^ Department of Plant Breeding and Genetics, Faculty of Agricultural Sciences, University of the Punjab, Lahore, Pakistan; ^2^ Department of Horticulture, Faculty of Agricultural Sciences, University of the Punjab, Lahore, Pakistan; ^3^ Department of Plant Pathology, Faculty of Agricultural Sciences, University of the Punjab, Lahore, Pakistan; ^4^ Department of Agronomy, Faculty of Agricultural Sciences, University of the Punjab, Lahore, Pakistan; ^5^ Department of Bioinformatics and Biotechnology, Government College University Faisalabad, Faisalabad, Pakistan; ^6^ Horticultural Sciences Department, University of Florida/Institute of Food and Agricultural Sciences (IFAS), North Florida Research and Education Center, Quincy, FL, United States; ^7^ Department of Zoology, College of Science, King Saud University, Riyadh, Saudi Arabia

**Keywords:** carotenoid cleavage oxygenases, apocarotenoids, RNA seq analysis, 9-cisepoxycarotenoid dioxygenases, *Oryza sativa*, *O. sativa*, abscisic acid, transcriptome

## Abstract

Rice constitutes a foundational cereal and plays a vital role in the culinary sector. However, the detriments of abiotic stress on rice quality and productivity are noteworthy. Carotenoid cleavage oxygenases (*CCO*) hold vital importance as they enable the particular breakdown of carotenoids and significantly contribute towards the growth and response to abiotic stress in rice. Due to the insufficient information regarding rice *CCOs* and their potential role in abiotic stress, their utilization in stress-resistant genetic breeding remains limited. The current research identified 16 *CCO* genes within the *Oryza sativa japonica* group. These Os*CCO* genes can be bifurcated into three categories based on their conserved sequences: *NCEDs* (9-Cis-epoxycarotenoid dioxygenases), *CCDs* (Carotenoid cleavage dioxygenases) and *CCD-like* (Carotenoid cleavage dioxygenases-like). Conserved motifs were found in the *OsCCO* gene sequence via MEME analysis and multiple sequence alignment. Stress-related cis-elements were detected in the promoter regions of *OsCCOs* genes, indicating their involvement in stress response. Additionally, the promoters of these genes had various components related to plant light, development, and hormone responsiveness, suggesting they may be responsive to plant hormones and involved in developmental processes. MicroRNAs play a pivotal role in the regulation of these 16 genes, underscoring their significance in rice gene regulation. Transcriptome data analysis suggests a tissue-specific expression pattern for rice *CCOs*. Only *OsNCED6* and *OsNCED10* significantly up-regulated during salt stress, as per RNA seq analyses. *CCD7* and *CCD8* levels were also higher in the *CCD* group during the inflorescence growth stage. This provides insight into the function of rice *CCOs* in abiotic stress response and identifies possible genes that could be beneficial for stress-resistant breeding.

## 1 Introduction

Carotenoids, these fascinating isoprenoid compounds, have captivated scientific curiosity ever since their serendipitous discovery in the 19th century (Stafsnes et al., 2010). Their enigmatic diversity unfolds through the analysis and identification of more than 700 distinct types over the years, revealing a rich tapestry of potential benefits waiting to be unraveled (Gitelson and Merzlyak, 1998). These compounds have various biological functions across different organisms, such as bacteria and plants (Misawa, 2011). Carotenoids serve as important secondary pigments in photosynthetic organisms, protecting them from photooxidation and helping to capture light more efficiently (Hashimoto et al., 2016). Additionally, certain apocarotenoids, produced through the cleavage of carotenoids, aid in regulating plant growth and development, as well as their response to biotic and abiotic stresses (Felemban et al., 2019). The carotenoid cleavage oxygenase’s (*CCOs*) are a series of enzymes involved in the breakdown of carotenoids which are naturally occurring pigments that contribute color to many fruits, vegetables, and flowers (Liang et al., 2018). *CCOs* comprise two types of enzymes carotenoid cleavage dioxygenases (*CCDs*) and 9-cis-epoxycarotenoid dioxygenases (*NCEDs*). These enzymes are essential for turning carotenoids into apocarotenoids, which have degraded (Yue et al., 2022). *CCOs* use oxygen to split the carotenoid molecule and produce apocarotenoids, which have a variety of physiological roles such as serving as plant hormones, enticing pollinators, and giving defense against environmental stress (Shi et al., 2023).

9-Cis-epoxycarotenoid dioxygenase (*NCED*) is a major enzyme in the ABA production pathway found in numerous plant species (Sun et al., 2012). *NCED* is evolutionarily conserved throughout plant lineages, with little divergence within its subfamily and excellent exon conservation (Liu et al., 2023). This demonstrates the significance of *NCED* in preserving the structural integrity of the protein for its function. *NCED* genes change somewhat slowly, which may contribute to the functional divergence of *NCED* subfamilies across tissues (Wan et al., 2021). Various expression patterns and activity levels of *NCED* subfamilies in various plant tissues may contribute to controlling ABA biosynthesis and plant responses to environmental signals (Pu et al., 2020).

The identification of the *NCED* gene marked a significant milestone in advancing our comprehension of ABA production and its role in plant growth and development. The *NCED* gene was initially cloned in the maize ABA-deletion mutant Vp14, leading to a better understanding of *NCED’s* involvement in ABA synthesis (Fei et al., 2023). Further research in Arabidopsis revealed nine *CCOs* genes, five of which *(AtNCED2, AtNCED3, AtNCED5, AtNCED6*, and *AtNCED9*) are involved in ABA synthesis (Hu et al., 2021). Manipulation of *AtNCED3* increased drought tolerance by increasing endogenous ABA accumulation (Seki et al., 2007). Identifying *NCED* genes in additional plant species, including Tamatim, cowpea, and rice, increased our understanding of *NCED* evolution and function across plant taxa (Wang and Guo, 2023). These findings show that *NCED* is a conserved and essential component of the ABA biosynthesis pathway that governs plant responses to environmental stress and developmental stimuli (Tan et al., 2003). The *NCED* gene family has been identified and investigated in many plant species, such as *Arabidopsis*  (Tan et al., 2003), citrus (Xian et al., 2014), cotton (Li et al., 2021), cucumber (Zhang et al., 2022), wheat, and tobacco (Zhang et al., 2014). However, the function of *NCED* genes in rice has not been clearly defined. Carotenoid cleavage dioxygenases (*CCDs*) are an enzyme that uses molecular oxygen to cleave carotenoids, a group of pigments commonly found in plants, animals, and microorganisms (Daruwalla and Kiser, 2020). CCDs are responsible for various biological functions such as vision, stress response, and reproduction, producing apocarotenoids with multiple biological activities (Wei et al., 2022). Arabidopsis now has nine *CCO* gene members, five *NCED* genes, and four *CCD* genes. The *CCD* gene family is divided into five subfamilies based on differences in cleavage sites and substrates: *CCD1, CCD4, CCD7, CCD8*, and *NCED* (Cheng et al., 2023). For the first time*, CCD-like (CCDL)*, a new member of the *CCD* family, was found in tomatoes (Cheng et al., 2022). The retinal pigment epithelium membrane protein (RPE65) domain is found in all *CCD* genes (Kiser, 2022), which is typical of enzymes involved in carotenoid cleavage (Kim et al., 2016). The *CCD* gene family has been found and studied in several plant species, including Arabidopsis (Chernys and Zeevaart, 2000). *CCD* gene family is described in grass species maize (*Zea mays*), rice (*O. sativa*), and sorghum (*Sorghum bicolor*) (Zhou et al., 2020). *CCD* research has also been conducted in wheat (Takezawa, 2000), watermelon (Cheng et al., 2022), pepper (Yao et al., 2022), tobacco (Zhou et al., 2019), rapeseed (Zhou et al., 2020), and maize (Sun et al., 2022). However, the function of *CCD* genes in rice has not been precisely defined. Extensive research on *CCO* genes has been conducted on Arabidopsis thaliana. Still, due to the absence of CCDL proteins, it is not an ideal candidate for investigating *CCD* enzymes and their functions. Fortunately, CCDL genes have been discovered *in Citrullus lanatus* and *Cucumis melo*, providing opportunities for further exploration (Xu et al., 2023). Carotenoids provide vibrant colors to fruits and vegetables, and their production and degradation are facilitated by *CCD* enzyme family (Kiokias et al., 2016). *C. lanatus and C. melo* have *CCDL* genes, making them good candidates for future research into *CCD* enzymes and their functions.

Rice (*O. sativa*) is a cereal crop grown in many parts of the world (Sami et al., 2023). It is a staple food for over half of the world’s population and is Asia’s most important food crop (Fukagawa and Ziska, 2019). The International Rice Genome Sequencing Project (IRGSP), a cooperation of research institutes from ten nations, sequenced the genome of rice *(O. sativa*) for the first time in 2002 (Project and Sasaki, 2005). However, research on rice *CCO* genes is sparse (He et al., 2022). The functional role of the *CCO* family in rice development remains unclear. To address this issue, we performed an extensive bioinformatics analysis of the complete rice genome in order to identify all potential members of the *OsCCO* gene family. We discovered several genes involved in the change in ABA content during rice seedling growth and development under environmental stress. The RNA-seq data analysis has identified promising candidates in *OsNCED6 and OsNCED10* genes, offering potential avenues for the development of salt stress-resistant rice varieties to enhance crop yield in saline conditions. Meanwhile, *OsCCD7 and OsCCD8* genes display higher activity in inflorescences, implying their potential involvement in grain development, although further investigations, such as gene cloning and functional analysis, are essential to validate their roles in diverse physiological and biological processes. This in-silico analysis expands our comprehensive understanding of the rice *OsCCO* gene family across the genome. The results of this study shed new light on the functional diversity and evolutionary aspects of the *CCO* gene family in plants. Our findings will serve as a valuable resource for further investigations, enabling the functional analysis and cloning of these genes through the comprehensive genome-wide identification and characterization conducted in this study.

## 2 Materials and method

### 2.1 Retrieve sequences from databases

The amino acid sequence data for *O. sativa* was obtained from the Phytozome v13 database (https://phytozome-next.jgi.doe.gov), and the particular domain PF03055 (RPE65) linked to the gene for carotenoid cleavage dioxygenase 1 (*CCD1*) was retrieved. The BLAST-P (Basic Local Alignment Search Tool for Protein Sequences) program was used to find the *CCD1* genes in the *O. sativa* genome database at Phytozome v13 using the PF03055 domain as a query. Using the default parameters, the obtained amino acid sequences were then cross-checked against NCBI’s Conserved Domain Database (*CDD)* (https://www.ncbi.nlm.nih.gov/genome/) (Zhang et al., 2016).

### 2.2 Physicochemical properties, subcellular localization and cis elements

Information on 16 protein was collected from Protparam (https://web.expasy.org/protparam/) and Phytozome. The Phytozome database provided crucial details such as chromosome number, location, and direction, mRNA length (CDS), and peptide length, while the Protparam database provided additional information including the theoretical pI (isoelectric point), molecular weight, GRAVY (Grand Average of Hydropathy) and stability index of the proteins. To identify the subcellular localization of the rice proteins, the WoLF PSORT database (https://wolfpsort.hgc.jp/) was utilized. This database employs protein sequences as input to generate a list of potential locations and their associated prediction scores. This investigation aimed to determine the most probable location of the proteins within the cell (Kavas et al., 2023). The upstream 1000bp promotor regions were extracted from phytozomeV3 (https://phytozome-next.jgi.doe.gov) for cis-regulatory elements extraction using a web tool PlantCARE (http://bioinformatics.psb.ugent.be/webtools/plantcare/html/). Putative cis-elements for the promotor region were retrieved from 5 to 20 bp. The results were visualized in a heatmap with the help of TBtools (Bülow and Hehl, 2016).

### 2.3 Analysis of conserved motif domain and exon-intron arrangement

The default values for the optimum matching length parameter were used in the Multiple Expectation Maximization for Motif Elicitation (MEME) program, which can be found online at (http://meme.sdsc.edu/meme/website/intro.html). Next, using the *CDD* search function, which discovers and curates domains identified by NCBI, the amino acid sequences were added to the Conserved Domain Database (*CDD*) (https://www.ncbi.nlm.nih.gov/Structure/cdd/wrpsb.cgi). In analyzing the distribution of exons and introns, the *OsCCO* gene family’s genomic and CDS sequences were subjected to the web tool Gene Structure Display Server (GSDS) at (http://gsds.cbi.pku.edu.cn/) (Barre et al., 2009).

### 2.4 Comparative phylogenetic analysis

*CCO* proteins’ amino acid (AA) sequences including *O. sativa, A. thaliana, S. lycopersicum, Citrullus lanatus, and Cucumis melo* were aligned to generate a phylogenetic tree. The tree was constructed using the neighbor-joining (NJ) method in the MEGA 11 software with 1000 replications for bootstrapping. The tree was further visualized and modified using the Interactive Tree of Life (iTOL) program (https://itol.embl.de/), which provided a user-friendly interface for exploring and annotating the tree (Rehman et al., 2022).

### 2.5 MiRNA analysis

The PmiREN website (https://www.pmiren.com/) was used to identify the target site of rice’s 16 *CCO* gene family. The PsRNA (https://www.zhaolab.org/psRNATarget/) online server tool was used to compare the CDS sequences of the genes with the mature miRNA sequences, and the default settings were utilized. The Cytoscape program was used to visualize the connections between target genes and the predicted miRNA (Mazhar et al., 2023).

### 2.6 Evolutionary analysis

The evolution of *CCO* genes in rice was examined using duplication and synteny analysis. The genes’ divergence period was estimated using Ka/Ks values. The protein sequences were aligned using the MUSCLE program, and for the calculation of Ka/Ks substitution rates, Tbtools 1.108 with default parameters was employed. This study calculated the molecular evolution rate for each gene pair by determining the Ka/Ks ratios of paralogous genes. To estimate the time of divergence between the paralogous, we used the Ks value in the T = Ks/2 equation, where λ equals 6.5 × 10^−9^. Gene duplication events were identified using MCScanX v1.0 with default settings. This analysis allowed us to determine the frequency of gene duplications and their potential impact on the molecular evolution of the paralogous genes. Tbtools were used to create syntenic and dual syntenic maps to ascertain the synteny link between Arabidopsis and tomato’s orthologous genes and rice’s paralogous genes (Mazhar et al., 2023).

### 2.7 Analyzing gene structure

The gene structure was analyzed using the gene structure display server (GSDS v2.0) (http://gsds.cbi.pku.edu.cn/) to identify intron-exon arrangements. PlantCare database (http://bioinformatics.psb.ugent.be/webtools/plantcare/html) was used to identify the cis-regulatory elements (Abdulla et al., 2023).

### 2.8 Gene ontology term analysis

Using GO annotations, a GO enrichment analysis was carried out to evaluate the roles of *CCO* genes in rice. Information on the molecular activities of the CCO genes and various biological processes was accessed from the Uniprot online database (https://www.uniprot.org/), which was consulted for this purpose (Langenbacher et al., 2020). The GO word enrichment analysis was then carried out using the *CCO* gene sequences entered into the ShinyGo v0.741 web tool, a resource accessible on (http://bioinformatics.sdstate.edu/go/) (Mazhar et al., 2023).

### 2.9 Protein-protein interaction

The activities of the CCO genes were further validated using GO annotations by gene ontology (GO) term enrichment study. The online program called ShinyGo v0.741.(https://string-db.org/cgi/input?sessionId=b16GBNb2GGmA) was used for better understand of CCO genes function in cucumber. For visualization of biological, molecular and cellular function, an online database shiny Go was used.

### 2.10 Expression analysis

#### 2.10.1 Gene expression profiling of salt-stressed rice plants

For this research, two types of rice plants (CSR28 and IR28) were selected to investigate their response to salt. Their data was retrieved from the NCBI GEO (https://www.ncbi.nlm.nih.gov/geo/) (GSE133480) database to explore the salt stress expression profile of the *CCO* gene family. The expression profiles of rice genotype were sub-categories into G1, G2, G3, G4, G5, G6, G7, and G8. Salt was applied to the roots and shoots of even genotypes for 6 hours. The second application of salt to the roots and shoots of genotypes with odd numbers for 54 hours. The Statistix 8.1 pairwise comparison tool was used to improve comprehension of up/down-regulated gene expression by highlighting significant differences between circumstances, assisting in the identification of possible targets for further research (Mazhar et al., 2023).

#### 2.10.2 Gene expression and inflorescence development in rice

Wild-type (WT) and Osgl1/2 mutant genotypes were analyzed for their inflorescence development stage in a study. Data from the NCBI GEO database (https://www.ncbi.nlm.nih.gov/geo/) (GSE227706) were used to evaluate the gene expression profiles of the *CCO* gene family. Statistix 8.1 pairwise comparison tool was used to determine significant differences in gene expression between the two genotypes, paving the way for further investigation into potential targets (Mazhar et al., 2023).

#### 2.10.3 Gene expression analysis of various part of rice

The gene expression profiles of the *CCO* gene family were assessed from the Expression Atlas database (https://www.ebi.ac.uk/gxa/experiments?experimentType=differential/). To gain insights into the expression patterns of these genes, RNA sequencing data were analyzed with the TBtools, which provides visual representations of gene expression levels.

## 3 Results

### 3.1 Identifying *CCO* genes in *O. sativa* and their localization

A total of 16 *CCO* genes have been identified in rice, with the encoding protein lengths ranging from 173 to 801 and molecular weights varying from 19,432.02 to 88,580.62 kDa. The smallest protein was *OsNCED4*, and the longest was *OsCCD9* (**Table 1**). Proteins were anticipated to be stable if their value was below 40 and unstable if it is above 40, a*cco*rding to the instability index. The genes *OsCCDL3, OsNCED4, OsCCD10, OsCCD13, OsCCD14*, and *OsNCED16* were predicted to be stable, while the remaining ten proteins, including *OsCCD1, OsCCDL2, OsCCD5, OsNCED6, OsCCD7, OsCCD8, OsCCD9, OsCCD11, OsCCDL12, and OsCCD15*, ware predicted to be unstable. The grand average of hydropathy (GRAVY) value suggests that most genes are predominantly hydrophobic, with *OsCCD1 and OsNCED4* showing a slight hydrophobic nature. The pI values for these proteins vary between 4.66 and 9.04, suggesting that they will carry no overall electrical charge within this pH range. Of the identified genes, 56.25% were in the forward direction, while the remaining 43.75% were in the reverse direction (**Table 1**).

**Table 1:** Physiochemical properties of *CCO* gene family.

*CCO* Gene ID	Accession	Chromosome		Direction	Size (AA)		pI	Mw (KD)	GRAVY	Instability index
	Phytozome V13	no.	Location		mRNA (CDS)	Peptide				
*OsCCD1*	LOC_Os10g08980.1	10	4857119.4859975	F	738	245	4.66	27827.49	0.171	49.26
*OsCCDL2*	LOC_Os08g28240.1	8	17229166.17235488	R	1725	574	6.19	65392.54	-0.268	46.29
*OsCCDL3*	LOC_Os08g28410.1	8	17338775.17348971	R	1437	478	5.87	54398.03	-0.213	35.09
*OsNCED4*	LOC_Os04g04230.1	4	1971151.1971673	F	522	173	4.97	19432.02	0.029	36.21
*OsCCD5*	LOC_Os04g46470.1	4	27567823.27570926	F	1830	609	9.04	68833.45	-0.295	51.65
*OsNCED6*	LOC_Os07g05940.1	7	2870685.2872832	F	1749	582	5.98	63733.36	-0.233	46.06
*OsCCD7*	LOC_Os01g38580.1	1	21665617.21669309	R	1659	552	5.73	60561.69	-0.188	50.25
*OsCCD8*	LOC_Os01g38580.2	1	21665617.21669309	R	1521	506	6.10	55517.02	-0.230	50.09
*OsCCD9*	LOC_Os01g54270.1	1	31220320.31228566	R	2406	801	5.79	88580.62	-0.259	46.69
*OsNCED10*	LOC_Os03g44380.1	3	24959106.24961777	F	1827	608	5.59	66437.10	-0.172	36.78
*OsCCD11*	LOC_Os02g47510.1	2	29026069.29028259	R	1917	638	5.93	69265.77	-0.148	41.95
*OsCCDL12*	LOC_Os09g15240.1	9	9251213.9255028	R	1647	548	5.68	61827.54	-0.181	40.88
*OsCCD13*	LOC_Os12g44310.1	12	27464734.27472036	F	1644	547	5.74	62352.21	-0.324	33.09
*OsCCD14*	LOC_Os12g44310.2	12	27464734.27472036	F	1623	540	5.70	61596.40	-0.311	32.73
*OsCCD15*	LOC_Os12g24800.1	12	14232902.14234903	R	1731	576	6.13	64514.10	-0.067	42.17
*OsNCED16*	LOC_Os12g42280.1	12	26268229.26270794	F	1842	613	5.64	66714.51	-0.140	38.62

**Figure 1:**
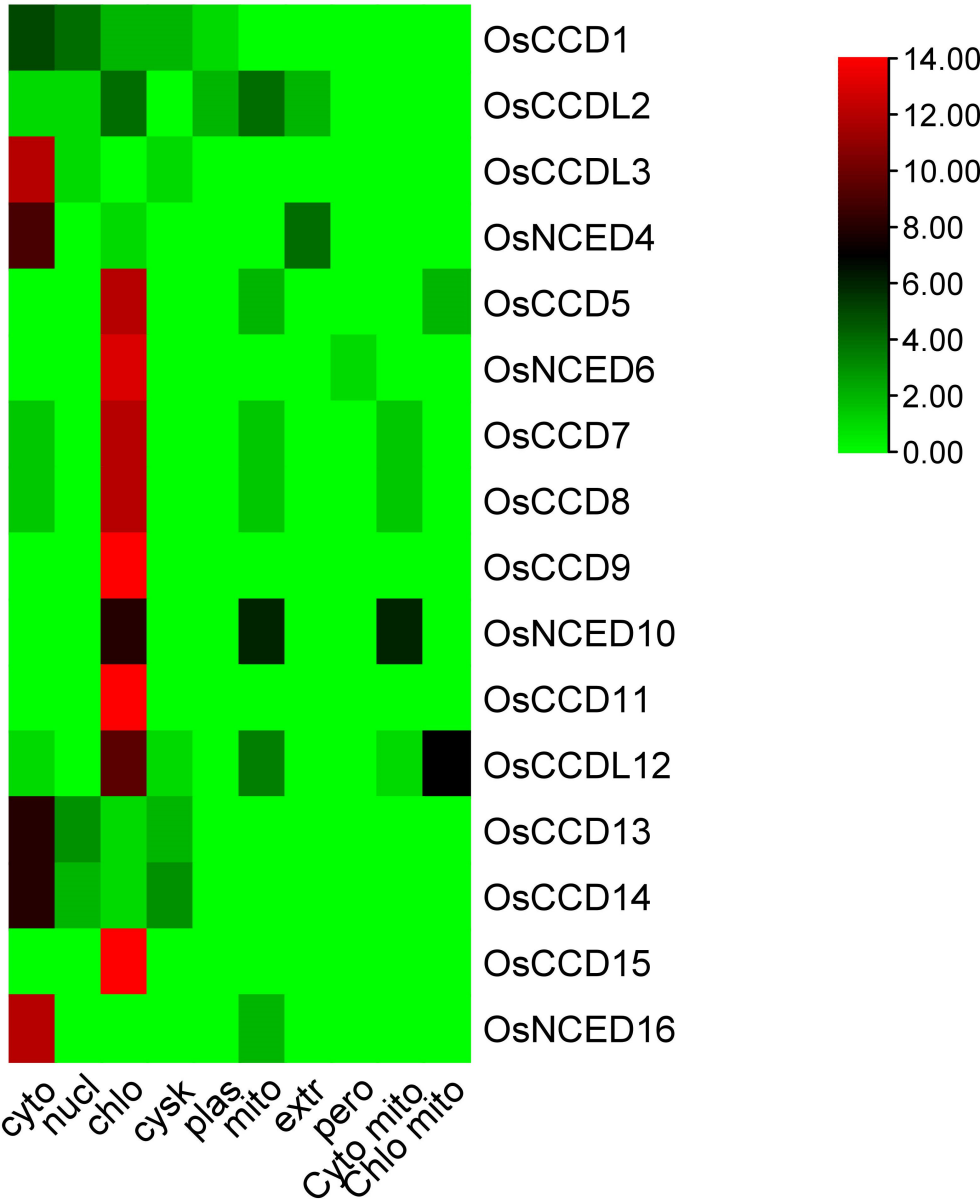
The subcellular localization prediction analysis of *OsCCO* proteins indicated their predominant localization in chloroplasts and cytoplasm. The analysis highlighted that the maximum number of proteins showed a red color, representing their localization in these cellular compartments.

The sub-cellular localization of these genes shows that *OsCCD1* was localized in both the cytoplasm and nucleus, while *OsCCDL2* was located in the chloroplast, mitochondria, and extracellular space.OsCCDL3 and OsNCED4 are located in the cytoplasm, while OsNCED5, *OsNCED6, OsNCED7, OsNCED8, OsNCED9, OsCCD11, OsCCD13, OsCCD14, and OsCCD15* were localized in the chloroplast. *OsNCED16* is also located in the chloroplast, while *OsNCED10 and* OsCCDL12 were localized in both the chloroplast and mitochondria (**Figure 1**; **Table 1**).

### 3.2 Conserved cis-elements

**Figure 2:**
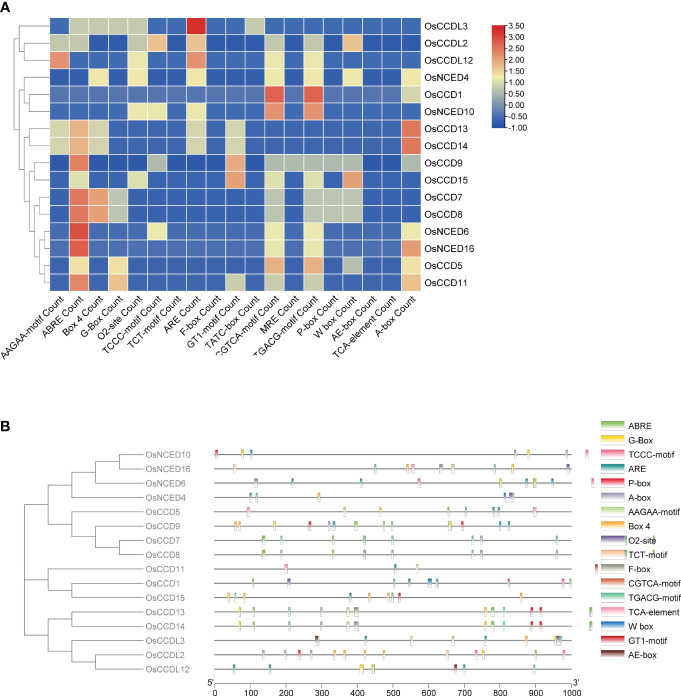
The analysis of cis-regulatory elements in the promoters of *OsCCO* genes in Rice revealed their association with various plant developmental processes. **(A)** The analysis provided statistics on the presence of cis-regulatory elements in each *OsCCO* gene. **(B)** The distribution of these cis-regulatory elements across the promoters was also investigated, shedding light on their spatial arrangement.

The analysis of *OsCCO* gene promoter regions identified 74 cis-elements, excluding common elements such as the TATA-box and CAAT-box, as well as unidentified functional elements. *OsCCO* genes in rice were subjected to cis-element analysis, and a variety of responsive elements were discovered, including 15 light-responsive elements, 28 stress-responsive elements, 19 development and metabolism-responsive elements, and 15 hormone-responsive elements. The light sensitivity of about 20.27% of the elements suggests that *OsCCO* genes may be involved in the response to light stress. The discovery that 37.83% of the cis-elements are linked to the stress response also suggests a likely role for *OsCCO* genes in stress-related activities. The study also identified 25.67% of the cis-elements associated with plant growth and metabolism, indicating that *OsCCO*genes may be involved in the growth and development of rice plants. The hormone-responsive cis-elements discovered (20.27%) include auxin, MeJA, and ABA. These elements may be interesting targets for study in order to understand how hormones behave under stress. All 16 *CCO* genes were found to contain most of the hormone-related regions. By employing molecular breeding techniques, the analysis provides valuable insights into the potential roles of *CCO* genes in rice, facilitating the creation of improved rice varieties (**Figure 2**; **Supplementary Table 1**).

### 3.3 Conserved motif analysis and domain prediction

**Figure 3:**
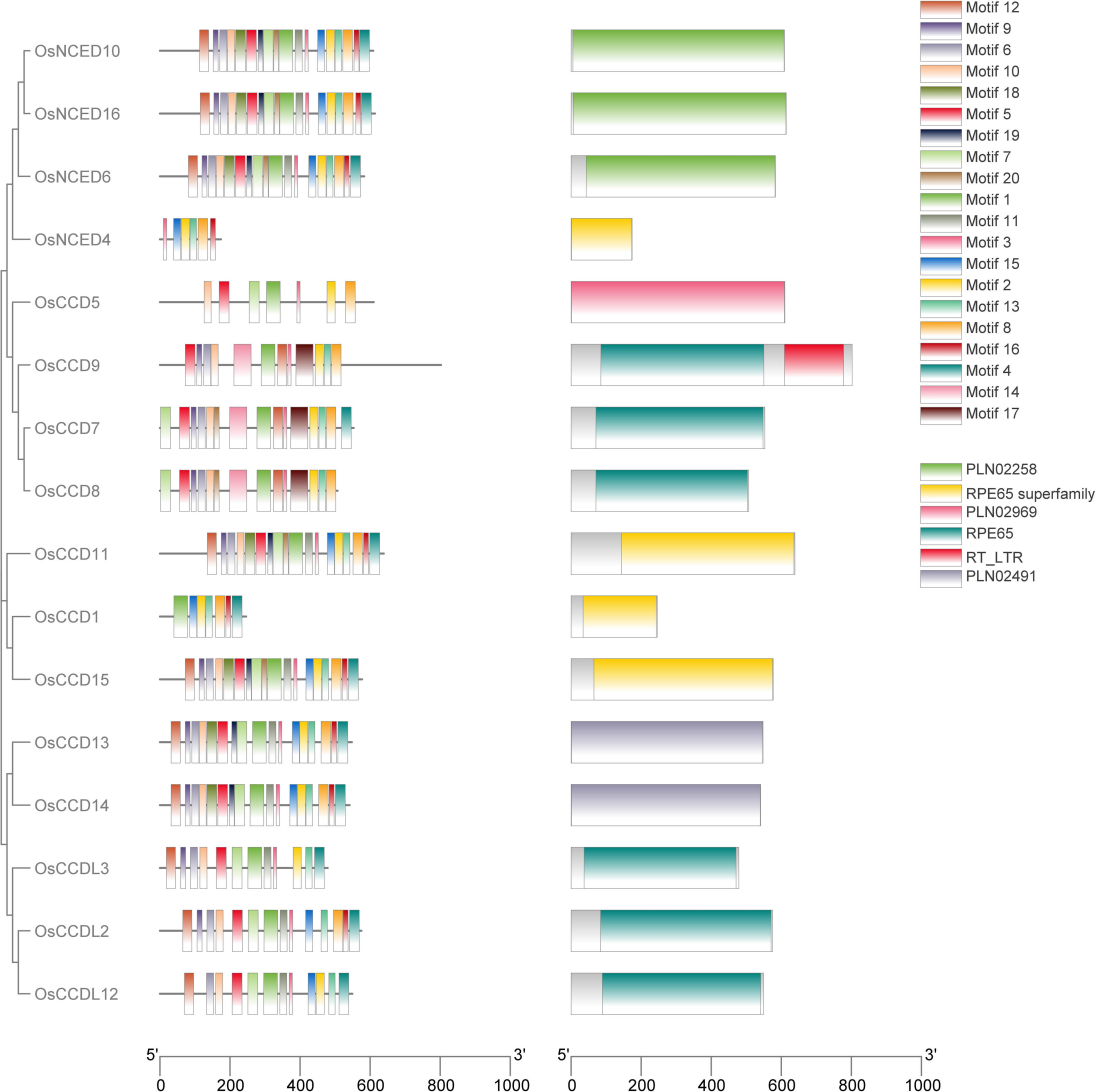
A bar graph with color-coded bars was generated to depict the motif distribution analysis of rice *OsCCO* proteins using MEME version 5.5.2. The analysis identified a total of 20 distinct motifs. To better understand the relationship between the *OsCCO* proteins and motif distribution, the graph was connected to a phylogenetic tree, providing additional insights into the evolutionary patterns and functional relationships among the OsCCO proteins.

The investigation carried out by the MEME tool on rice *OsCCO* looked into the discovery and distribution of 20 distinct motifs. Among all the *OsCCO* proteins, RPE65 superfamily, RPE65, PLN02258, PLN02969 domains were present (**Supplementary Figure 1**; **Supplementary Table 2**). During conserved domain investigation the OsCCD1, OsNCED4, OsCCD11, and OsCCD15 protein were categorized under the RPE65 superfamily. Within the same RPE65 superfamily the RPE65 were consisted of OsCCDL2, OsCCDL3, OsCCD7, OsCCD8 and OsCCDL12. Notably, OsCCD9 was associated with both the RPE65 and RT_LTR subfamilies’ While OsNCED6, OsNCED10 and OsNCED16 were grouped under the PLN02258 classification. In a separate classification, OsCCD5 was linked to the PLN02969 subfamily. OsCCD13 and OsCCD14 were both members of the PLN02491 subfamily. So it was cleared that domain was conserved throughout all proteins.While analyzing the *OsCCO* gene family, it was discovered that genes within the same group shared similar motifs. This suggests that conserved motifs might play a role in specific activities within a particular group or subgroup. During conserved motif investigation, the motif (2, 8, 13, 15, and 16) were conserved in the RPE65 superfamily. While RPE65 had a conserved motif of motif (1, 3, 5, 6, 7, 10, 12 and 13). While motif (1, 2, 3, 4, 5, 6, 7, 8, 9, 10, 11, 12, 13, 15, 16, 18, 19 and 20) were conserved in the PLN02258. The motif (1, 2, 3, 4, 5, 6, 7, 8, 9, 10, 11, 12, 13, 15, 16, 18 and 19) were conserved in the PLN02491 subfamily (**Supplementary Figure 2**). Notably, RPE65 and RT_LTR subfamilies’ were consist of motif (1, 2, 3, 5, 6, 8, 9, 10, 12, 13, 14, and 17), while PLN02969 subfamily was consist of motif (1, 2, 3, 5, 7, 8, and 10) (**Supplementary Figure 2**; **Supplementary Table 3**). The presence of similar motifs across different members of the *OsCCO* gene family hints that gene expansion might have played a role in the evolution of these genes. Unquestionably, OsNCED4, OsCCD5, and *OsCCD1* had fewer motifs like 6, 7, and 7, respectively. This study discovered that none of the 16 genes analyzed had any conserved motifs. However, 15 genes have a conserved motif, indicating a possible functional significance (**Figure 3**).

### 3.4 Exon intron analysis

**Figure 4:**
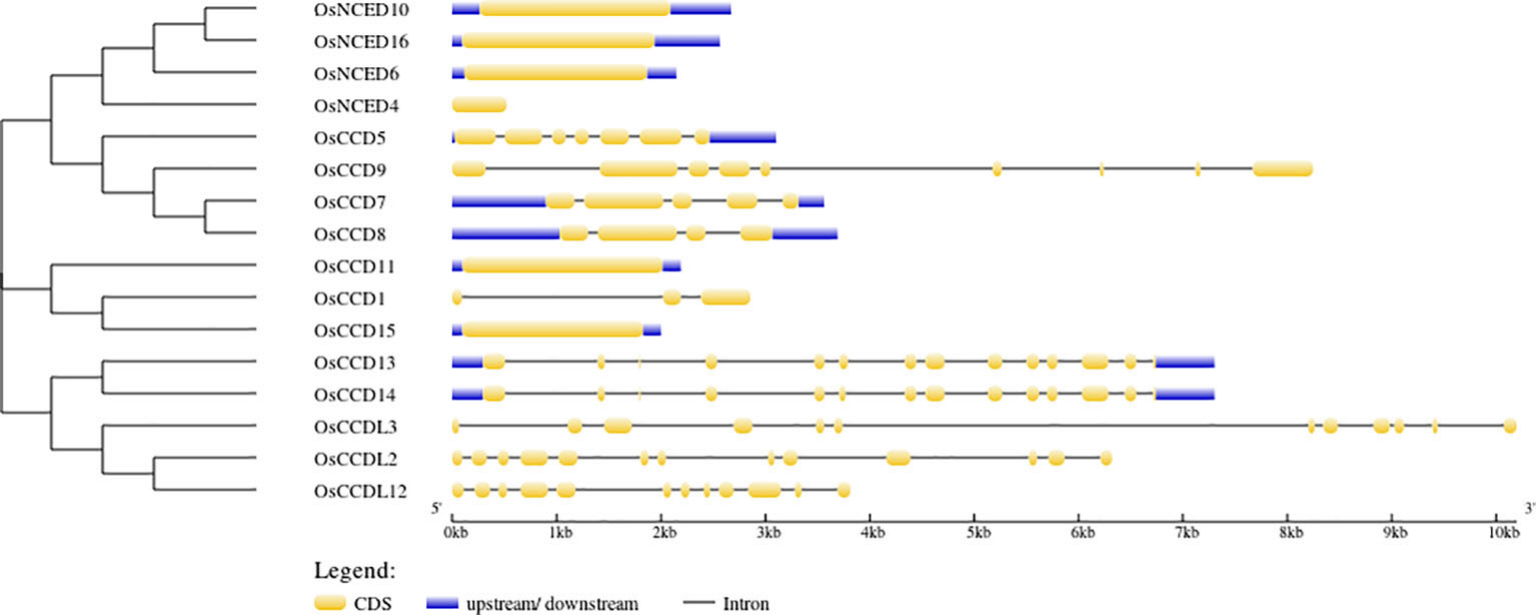
The intron-exon structures were illustrated using yellow and black shapes, representing exons within the genes.

According to gene structure predictions*, 6 OsCCO* genes (37.5%) were found to have only one exon and no introns. This indicates that these genes have a simple structure with no gaps in their coding sequence. Three genes (18.75%) had 13 exons and 12 introns, while 2 genes (12.5%) had 12 exons and 11 introns. There are 19 cis-elements found in the *CCO* gene (**Figure 4**).

### 3.5 Analysis of the *CCO* gene family’s phylogeny

**Figure 5:**
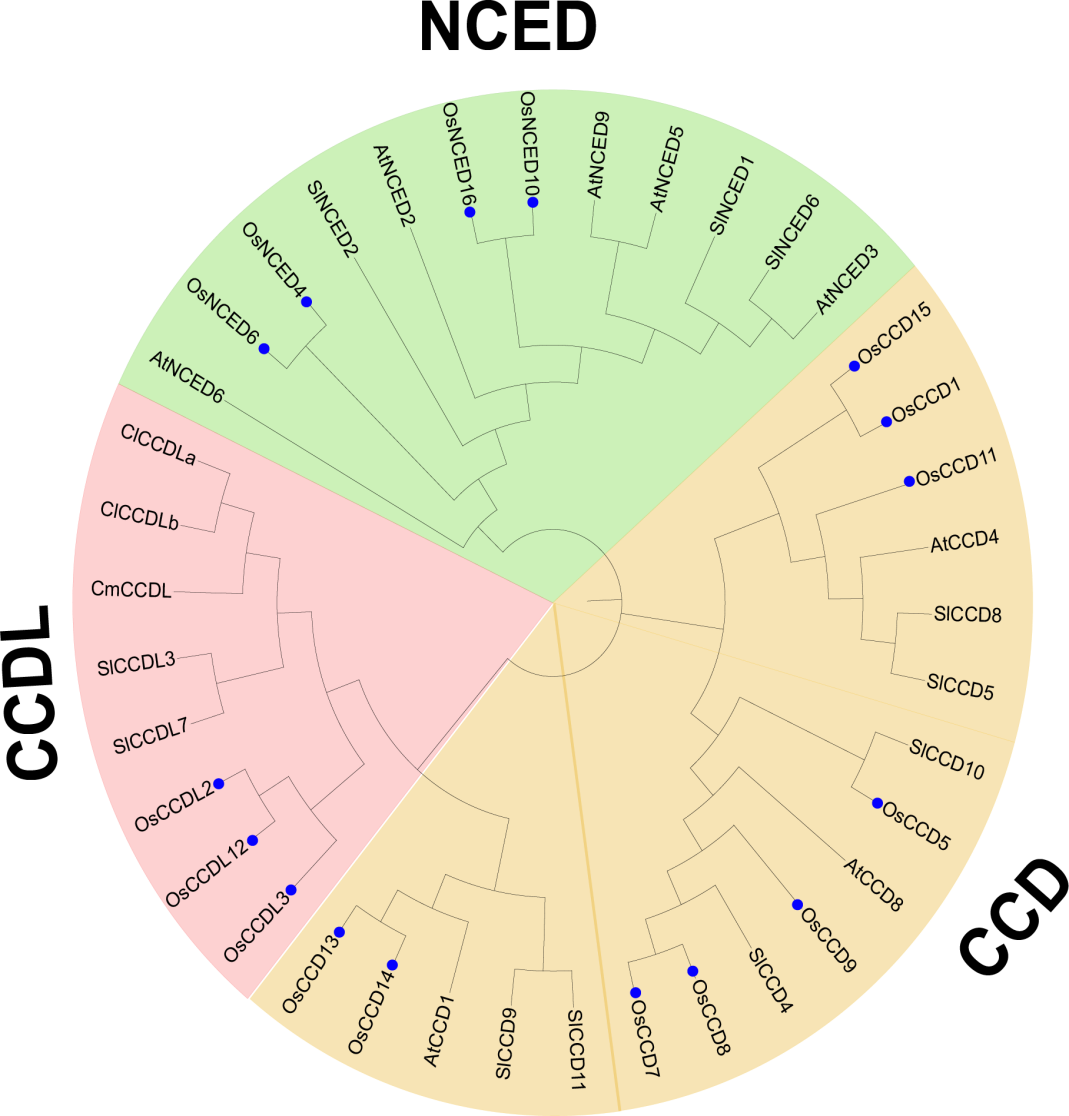
Evolutionary analysis was conducted to investigate the phylogenetic relationship among the CCO gene family members of *O. sativa*, *Citrullus lanatus*, *Cucumis melo*, *Arabidopsis thaliana*, and *Solanum lycopersicum*. The O. sativa genes were specifically identified using blue stars. The analyses were performed using MEGA 11 software, allowing for comprehensive insights into the evolutionary patterns and relationships among these species’.

The phylogenetic analysis revealed that the *CCO* genes from five plant species were classified into three subgroups, denoted as subfamilies I-III. Specifically, the study analyzed 37 *CCO* genes, including 16 from *O. sativa*, 11 from *S. lycopersicum*, 8 from *Arabidopsis thaliana*, and 1 from and *Cucumis melo*. To better understand the properties of subfamily III, represented by *CCDL*, sequences from *C. lanatus and C. melo* were added to the analysis. *CCDL* is noticeably absent in Arabidopsis thaliana, so genomes from several additional plant species were inserted. In the resulting phylogenetic tree, *O. sativa CCO* genes were marked by blue dots, and subfamilies were differentiated by color coding, with subfamily I represented by green, subfamily II by blue, and subfamily III by pink (**Figure 5**).

### 3.6 MicroRNA analysis

A total of 153 miRNAs were identified that target all 16 genes. These 153 miRNAs belong to 45 groups of miRNAs. The length of these miRNAs ranges from 20 to 24 amino acids. *OsCCDL12* (Osa-miR169, Osa-miR5802, Osa-miR1430, Osa-miRN2366, Osa-miR1432, Osa-miRN2322) is targeted by 6 groups of miRNAs, which is the highest number of targets, while *OsCCD1* (Osa-miRN2275) is targeted by only 1 miRNA. *OsCCD7* and *OsCCD8* are targeted by 4 and 2 groups of miRNAs, respectively. OsCCDL3 and *OsNCED6* are targeted by 4 and 1 group of miRNAs, respectively. *OsCCD13* and *OsCCD14* are targeted by 3 and 2 groups of miRNAs, respectively. *OsCCD11*, *OsCCD15*, *OsCCD5*, *OsCCD9*, *OsNCED10*, and *OsNCED16* are targeted by 4, 3, 3, 4, 3, and 1 group of miRNAs, respectively. The miRNAs are specific and target only one gene, but multiple miRNAs can target a single gene. Most of the miRNAs inhibit cleavage, while others inhibit the translation of their respective targeted genes. In total, 49 groups consist of 12 translation-inhibited miRNAs and 37 cleavage-inhibited miRNAs found in the results. Osa-miR169, Osa-miR827, Osa-miR394, Osa-miR395, Osa-miR396 and Osa-miR397 were involved during salt in rice (Wang et al., 2013) (**Supplementary Table 4**).

### 3.7 *CCO* gene duplication and Sydney analysis

**Figure 6:**
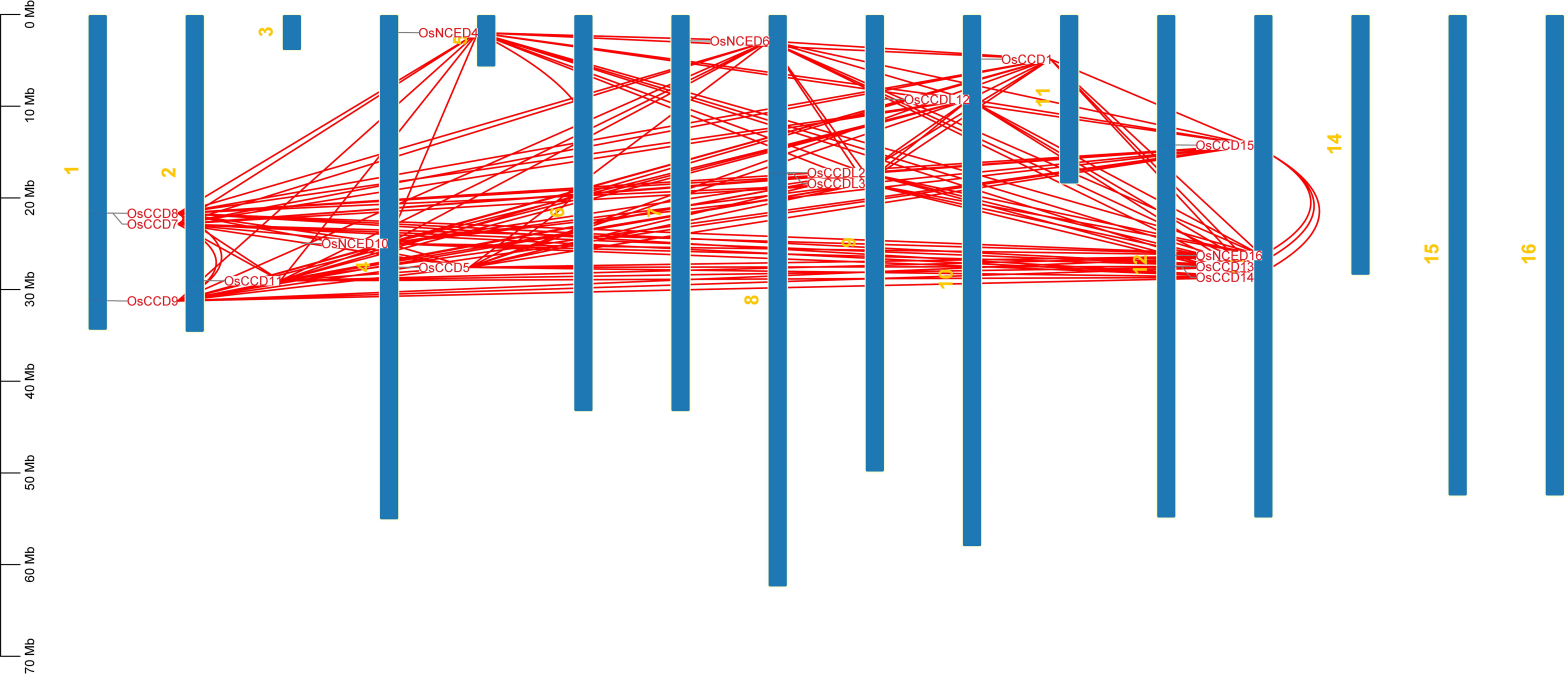
The chromosomal distribution of the *OsCCO* gene family in rice is illustrated in the figure. The figure’s blue bars represent rice’s individual chromosomes, while the red lines indicate the functional relationships or co-regulation between *OsCCO* genes. This analysis provides insights into the spatial arrangement and potential interactions among *OsCCO* genes within the rice genome.

Chromosomal location anticipated that they were dispersed on distinct chromosomes. *CCO* genes were found on chromosomes 1, 2, 3, 4, 7, 8, 9, 10, and 12. No *CCO* genes exist on chromosomes 5, 11, 13, 14, or 16. *OsCCDL2* and *OsCCDL3* are found on chromosome 8. *OsCCD7*, *OsCCD8, and OsCCD9* were also found on one chromosome*. OsNCED4* and *OsCCD5* were discovered on four chromosomes*. OsCCD13, OsCCD14, OsCCD15*, and *OsNCED16* are all found on chromosome 12 (**Figure 6**).

**Figure 7:**
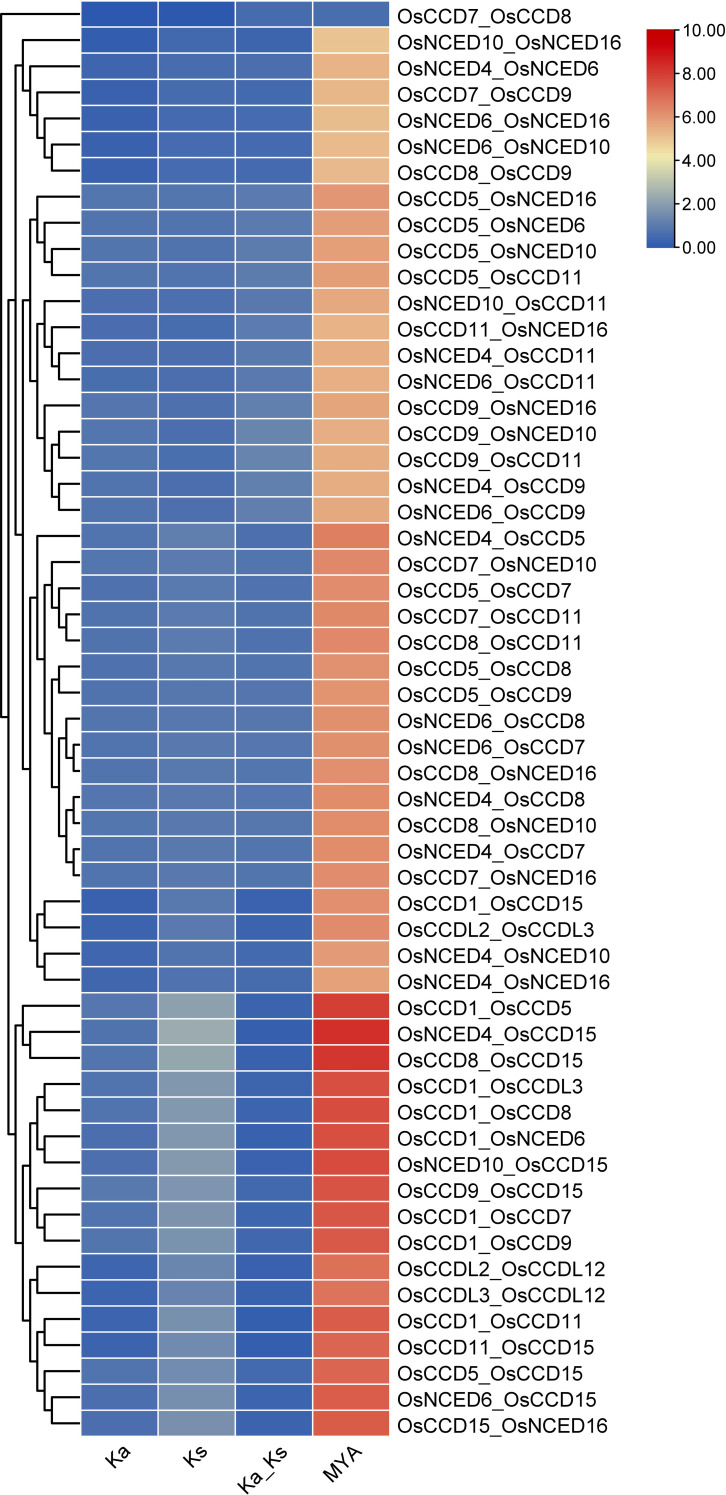
The Ks (synonymous substitution rate) and Ka (nonsynonymous substitution rate) were estimated using TBTools. The rectangular-like rate (λ) for rice was calculated to be 6.5 × 10−9. The date of the duplication event was determined using the formula T = Ks/2λ. This analysis provides insights into the timing and evolutionary dynamics of gene duplications in rice.

For the gene pair *OsCCD7_OsCCD8*, the Ka and Ks values are relatively low (0.0037 and 0.0073, respectively), indicating that the genes have experienced relatively few substitutions. The MYA value of 0.56 suggests that the gene divergence occurred relatively recently. While for the gene pair *OsNCED4_OsCCD15*, the Ka and Ks values are much higher (0.72 and 4.18, respectively), indicating that these genes have experienced many substitutions over time. The MYA value of 321.65 suggests that these genes diverged from a common ancestor long ago. The two gene pairs have undergone different levels of evolutionary change, with *OsNCED4_OsCCD15* experiencing more divergence over time than *OsCCD7_OsCCD8* (**Figure 7**).

**Figure 8:**
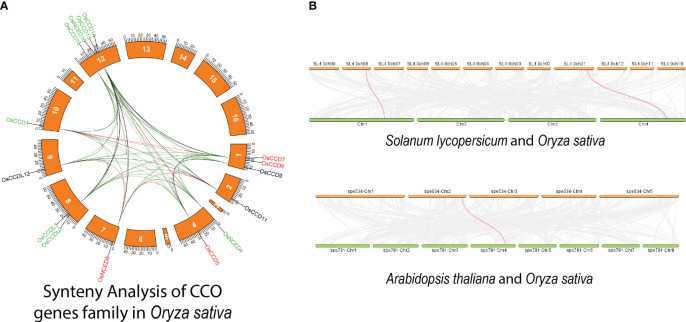
**(A)** Displays the distribution of *OsCCO* genes on rice chromosomes, where potential gene duplications are indicated by lines connecting genes on different chromosomes. Panel **(B)** shows the chromosomal distribution and intrachromosomal linkages of *CCO* genes between rice-Arabidopsis and rice-Tomato. Red lines represent duplicate *CCO* gene pairs, while gray lines represent synteny blocks in the rice genome. The chromosome number is indicated at the top of each chromosome, highlighting that segmental duplication of genes is more prevalent than tandem duplication within the *OsCCO* gene family. This analysis provides insights into the evolutionary history and genomic organization of *OsCCO* genes.

During synteny analysis, 56 paralogous genes were found, but the *OsNCED10* gene was a singleton, meaning no copies were discovered (**Figure 8A**). One gene was duplicated in Arabidopsis, however two duplicate genes were found in rice and tomato, according to the dual synteny study (**Figures 8A, B**).

### 3.8 Go annotation and orthologue identification

**Figure 9:**
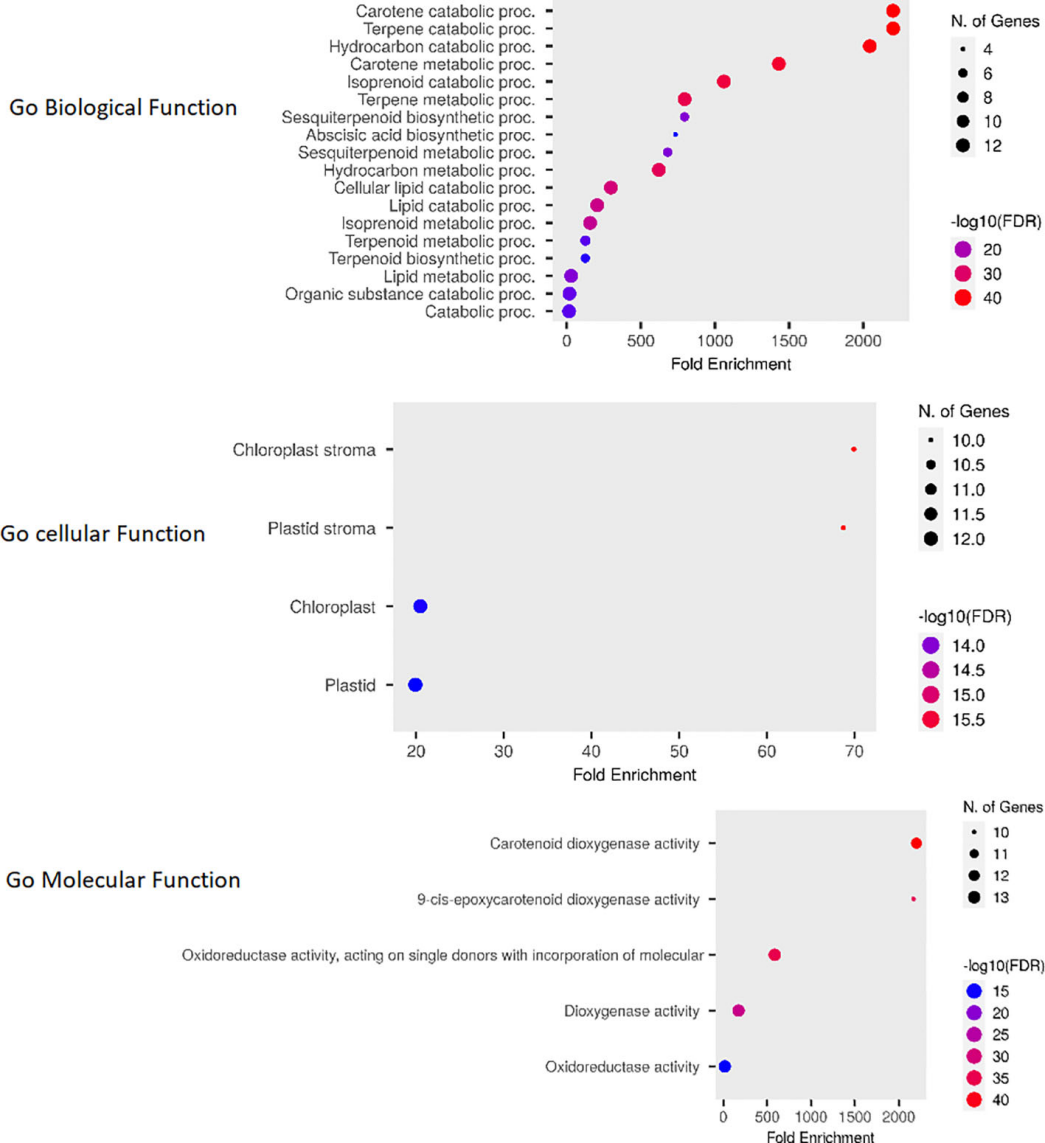
The Fold Enrichment chart visually represents the overlapping functions of *OsCCO* genes. The chart uses dot plots, where red dots indicate a higher number of genes associated with a particular process, while smaller blue dots represent a lower number of genes. This chart provides a quick overview of the functional distribution among *OsCCO* genes and highlights the processes in which they are predominantly involved.

The Go enrichment analysis conducted in this study revealed valuable insights into the functional roles of *OsCCO* genes. These genes were found to be primarily involved in carotene metabolic processes (GO:0016119) and terpene catabolic processes (GO:0046247) within the biological process category (Rothenberg et al., 2019). In terms of molecular functions, they were prominently associated with carotenoid dioxygenase activity (GO:0010436). Furthermore, the analysis indicated a high enrichment of *OsCCOs* proteins in the chloroplast stroma (GO:0009570), suggesting their diverse roles in cellular metabolism (**Figure 9**; **Supplementary Table 5**).

### 3.9 Protein interaction

**Figure 10:**
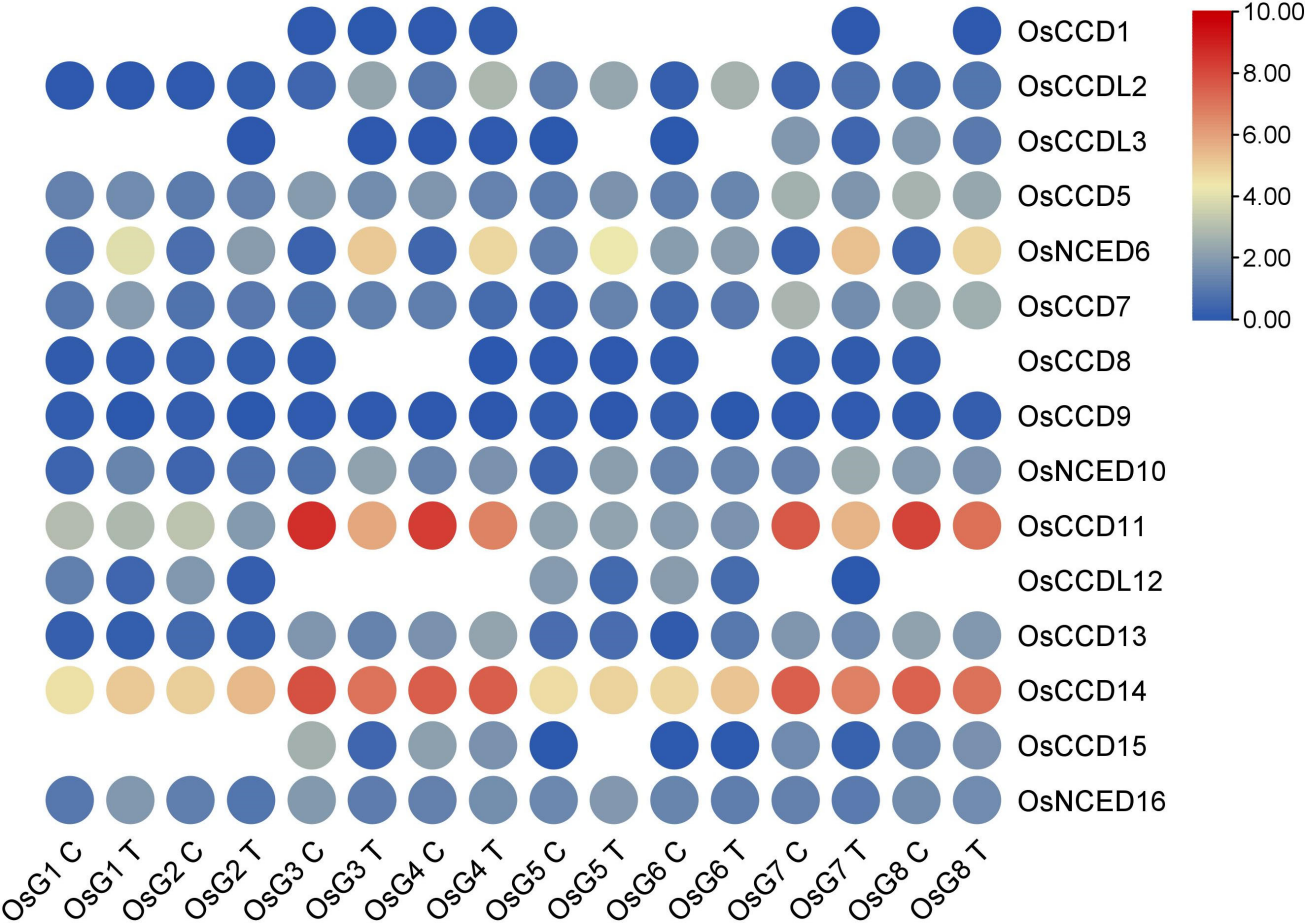
Protein-protein interactions involve various connections with distinct genes.

The *OsCCD11* gene exhibits the highest number of associations in the protein-protein interaction network. Notably, *OsCCD11, OsNCED4, OsNCED6, OsNCED16, and OsCCD1* establish distinctive connections among themselves. Conversely, *OsCCDL12 and OsCCDL3* engage with a diverse set of proteins in the dataset, indicating their broad interaction profiles. *OsCCD11* appears to participate in various connections and play roles in multiple cellular processes. Meanwhile, *OsCCD8, OsCCD9, and OsCCD5* form a sub-network within the larger protein-protein interaction network, suggesting a specific biological process or coordinated functional relationship among these proteins (**Figure 10**).

### 3.10 Transcriptomic analysis

#### 3.9.1 Gene expression profiling of salt-stressed rice plants

**Figure 11:**
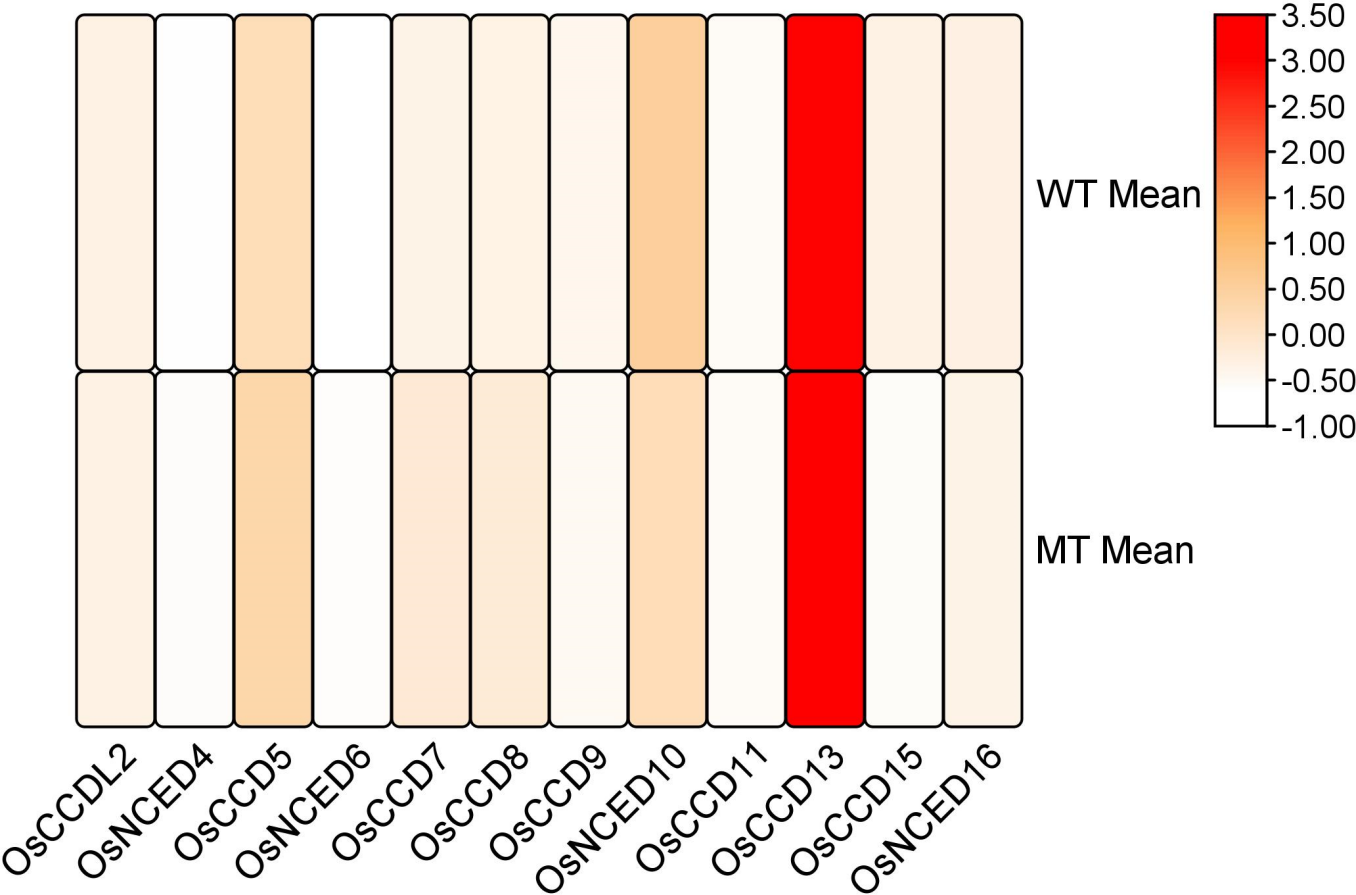
Heat map of transcriptomic expression profile of the *OsCCO* genes of rice under salt conditions.

Based on the RNA-seq analysis, it was observed that *OsNCED10 and OsNCED6* showed high expression levels and upregulation, indicating their potential role in abscisic acid (ABA) production. This suggests these genes regulate ABA synthesis in rice plants, especially in response to stress conditions. However, based on their expression patterns, the remaining genes did not exhibit significant roles in salt stress (**Figure 11**).

#### 3.9.2 Gene expression and florescence development in rice

**Figure 12:**
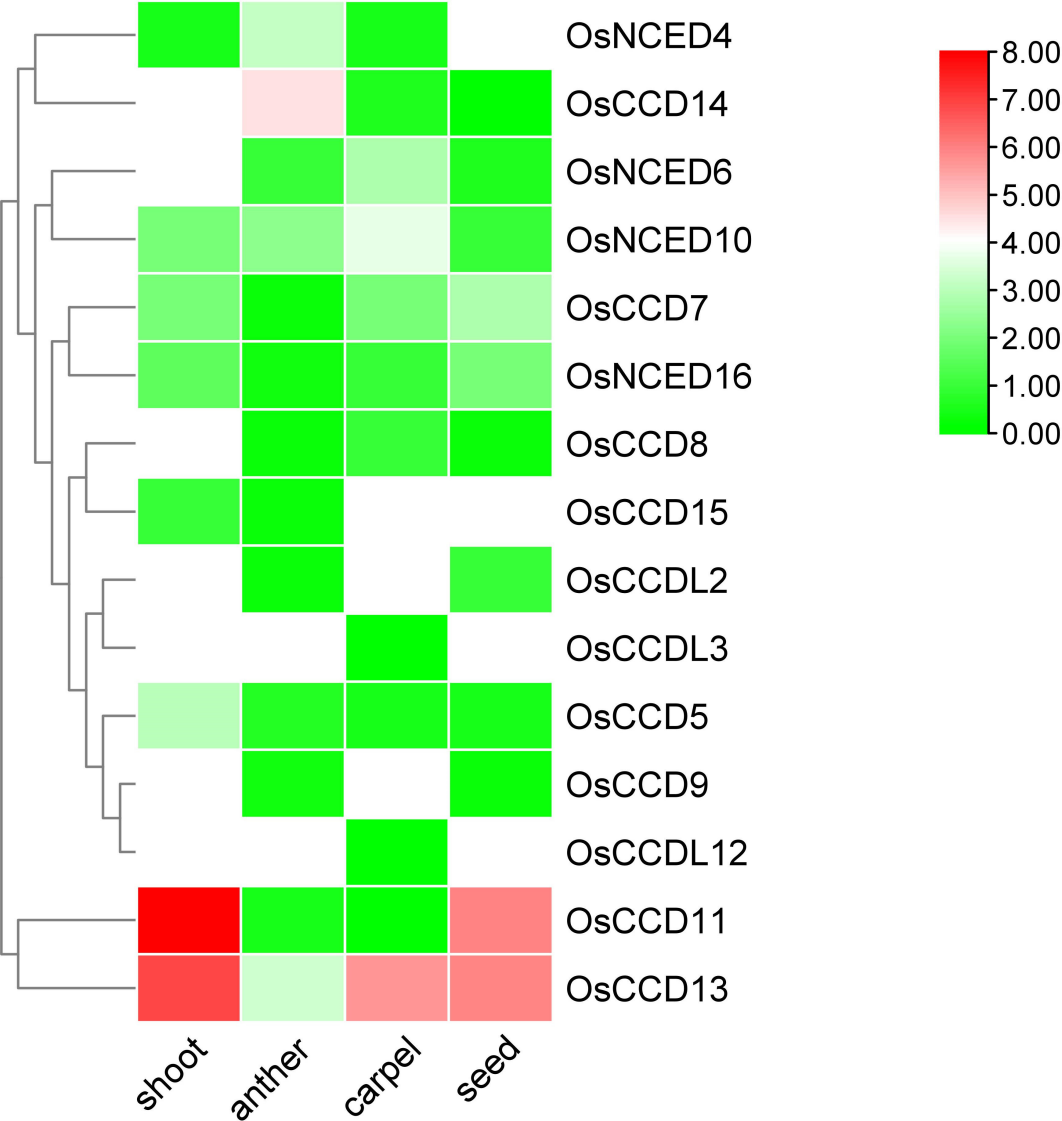
Heat map of transcriptomic expression profile of the *OsCCO* genes during inflorescences development.

RNA-seq analysis revealed that during the inflorescence development stage, the mutant genotype exhibited high expression levels and upregulation of two genes, *CCD7 and CCD8*. These genes showed significant involvement in the developmental process. However, the remaining genes were also expressed during the inflorescence development stage but did not display a significant role (**Figure 12**).

#### 3.9.3 Gene expression analysis in various part of rice

RNA-seq analysis revealed distinct expression patterns of various genes. *OsCCDL*2 was predominantly expressed in seed, while *OsCCDL3* showed expression specifically in carpel tissue. *OsNCED4* exhibited high expression levels in anther tissue, while *OsCCD5* was highly expressed in shoot tissue. *OsNCED6* showed upregulation in carpel tissue, whereas *OsCCD7* displayed upregulation in seed and expression in carpel and shoot tissues. *OsCCD8* was expressed in carpel tissue, and *OsCCD9* showed expression in anther and seed tissues.

**Figure 13:**
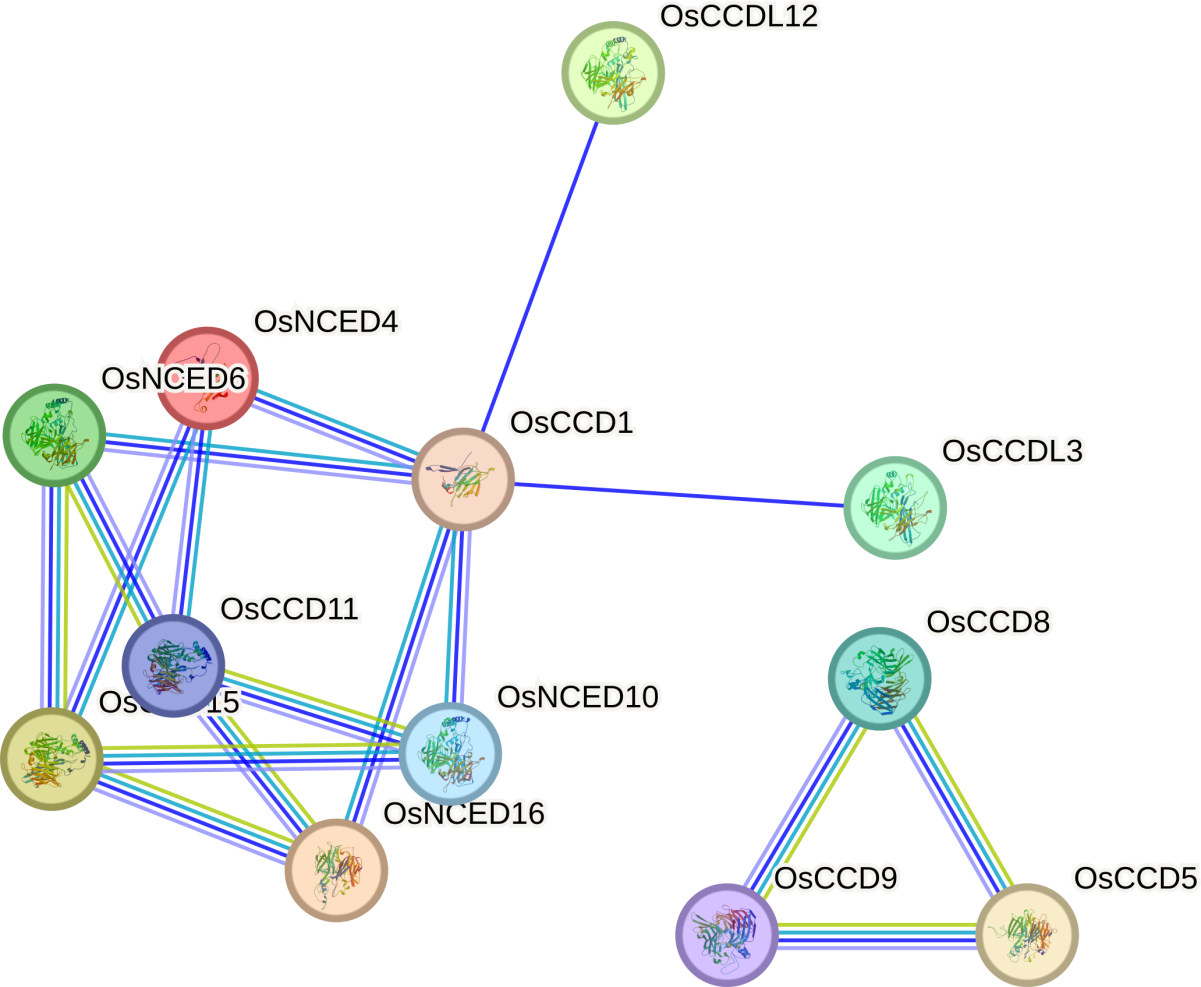
The heat map visually displays the transcriptomic expression patterns of *OsCCO* genes in different organs of rice. It uses colors to represent the varying levels of gene expression.

Furthermore, *OsNCED10* was highly upregulated in carpel tissue, indicating its potential role in this tissue-specific process. *OsCCD11* exhibited high upregulation in shoot and seed tissues. *OsCCDL12* was expressed in carpel tissue, while *OsCCD13* showed high upregulation in shoot, seed, and carpel tissues. *OsCCD14* displayed significant upregulation in anther tissue, and *OsCCD15 showed* expression specifically in shoot tissue. Lastly, *OsNCED16* exhibited high upregulation in seed and shoot tissues (**Figure 13**).

## 4 Discussion

Rice is a major crop for many poor countries, but because of many environmental stressors, both abiotic and biotic, it is vulnerable to yield reductions (Seah et al., 2023). To help minimize these losses, transcription factors (TFs) play a vital role in regulating plant responses to these stresses. The investigation of *OsCCOs* gene family is essential in plant research particularly in relation to its role in environmental stress such as drought, seed dormancy, salinity, cold and heat stress. The role of the *OsCCOs* gene family in rice is yet to be fully comprehended. Knowledge of *CCO* genes’ functions and their association with these activities opens the door for novel approaches to improve crop production and increase the resilience of rice plants against environmental stressors (Dhankher and Foyer, 2018).

A genome-wide investigation was carried out to find and understand the *CCO* genes in rice (Griffiths et al., 2003). The physicochemical properties of sixteen *OsCCO* genes in the rice genome were investigated to observe their distinctions within a clade of proteins. It was determined that all identified *OsCCO* proteins exhibited hydrophilic characteristics as evidenced by negative GRAVY values, reflecting a tendency to interact with water and possess electrical charges dependent on pH levels (Wilkins et al., 1998). Upon examination using the instability index, it was observed that ten distinct proteins possessed features suggestive of instability. The examination of subcellular localization demonstrated that *OsCCO* proteins were dispersed among multiple organelles, such as the chloroplast, mitochondria, cytoplasm, cytosol, endoplasmic reticulum nucleus and plasma membrane (Tan et al., 2003). It is significant to observe that more than half of the proteins, precisely 47.76% and 23.98%, were correspondingly identified in the chloroplast and cytoplasm. This suggests that the *OsCCO* proteins could serve vital roles within these particular organelles.

By examining the genome of diverse species, substantial insight can be gained regarding the history and arrangement of genes. Moreover, such analyses can enable the transfer of genomic data from a well-researched taxonomic group to one that is not as extensively studied (Auldridge et al., 2006). In the current research, 56 paralogous genes were detected within *OsCCO*, indicating genes replicating via gene duplication (Xue et al., 2023). This occurrence of duplication grants particularly valuable knowledge into the expansion of gene families, a phenomenon frequently observed in the realm of plants as a result of both tandem and segmental duplications (Fatima et al., 2023).

Comparative analysis of members of similar subgroups can provide significant insights into their functional behavior. This study, 37 *CCO* proteins were discovered and classified into three subfamilies based on their sequence structures and evolutionary relationships (Xue et al., 2023). Twelve *OsNCED* proteins, seventeen *OsCCD* proteins, and eight *OsCCDL* proteins were discovered using phylogenetic analysis. These findings imply that the *OsNCED* and *OsCCD* proteins in their respective subgroups may perform roles similar to *AtNCED* and *AtCCD* in Arabidopsis. In contrast, the *OsCCDL* proteins may perform functions similar to *ClCCDLa*, *ClCCDLb*, and *CmCCDL* proteins (Zhao et al., 2022).

Previous studies have suggested that positioning exons and introns within gene families is important for evolution (Zhao et al., 2022). This study’s investigation of gene structure and motifs revealed that the distribution of exons, introns, and motifs among members of the same population and clade was compatible with the shape of the phylogenetic tree (Wei et al., 2022). Every *CCO* gene contained exons and introns (**Figure 4**). Notably, in keeping with the traits of plants, the motifs of the *NCED* subfamily were found to be more maintained than those of the *CCD* subfamily.

Cis-regulatory elements are frequently located in the promoter region of genes and are essential for controlling gene expression at the transcriptional level (Zhou et al., 2019). Research on cis-regulatory elements revealed that a significant portion of the largest group, constituting 28 elements (equivalent to 37.833%), had a stress response and contained motifs such as ABRE, MYB, STRE, TGACG-motif, and WRE3. Meanwhile, the second-largest group, composed of 19 elements (or 25.67%), focused on metabolism and development and contained motifs such as A-box, Box 4, and TCCC-motif (Zhou et al., 2019). In addition, *OsCCO* contains various motifs responsible for different responses, such as CGTCA-motif and TGACG-motif for the MeJA response, TCA-element for the SA response, GARE-motif, TATC-box, and P-box for the GA response, and the ABRE TGA-element for the Auxin response.

Plants can undergo transpiration and photosynthetic processes, which can be affected by drought and salinity, resulting in reduced crop yields. The role of stomata is crucial in this process (Ahluwalia et al., 2021). Rice plants under salt stress were examined for the functions of various genes, including *OsCCO* genes, using transcriptome data (Liang et al., 2021). Interestingly, while *CCD* genes did not show any impact during salt stress, *OsNCED6* and *OsNCED10* genes were identified as possible candidates for developing salt-resistant rice varieties based on the RNA-seq data analysis. By producing ABA hormone, these genes facilitate stomata closure, resulting in reduced water loss due to transpiration and improved water-use efficiency under salt-stress conditions, thereby impacting rice Physiology and development.

The Go enrichment analysis of *OsCCO* genes highlights their significant involvement in carotene metabolic and terpene catabolic processes (Bao et al., 2021), pointing to their roles in pigment synthesis, vitamin A production and plant defense mechanisms. Additionally, their association with carotenoid dioxygenase activity underscores their potential in carotenoid cleavage reactions. The high enrichment of *OsCCO* gene in the chloroplast stroma suggests their crucial participation in diverse metabolic processes within this organelle, particularly related to photosynthesis and pigment biosynthesis (Chengcheng et al., 2022).

OsCCD11 stands out with the highest number of associations in our network analysis. This protein, along with OsNCED4, OsNCED6, OsNCED16, and OsCCD1 forms a distinct cluster of interconnected proteins, implying their potential involvement in shared cellular processes. Conversely, OsCCDL12 and OsCCDL3 exhibit a broader range of interactions, suggesting their roles in diverse biological contexts. These results hint at a specific functional relationship or concerted involvement in a particular biological process within the larger interaction network (Shimada et al., 2017).

Through a comprehensive transcriptome analysis utilizing RNA sequencing data from the NCBI GEO database (GSE227706), the importance of the *CCD* group in rice has been elucidated (Zhao et al., 2021). An in-depth investigation unveiled that *CCD7 and CCD8* exhibited elevated expression levels during the critical floral development phase in rice. This significant discovery not only opens doors for further research aimed at enhancing rice productivity and quality but also establishes a potential foundation for the development of novel rice varieties with enhanced traits (Yue et al., 2022).

The analysis of RNA-seq data of various part of rice gene expression investigations (available at https://www.ebi.ac.uk/gxa/experiments?experimentType=differential/) has revealed important information about the expression patterns of *OsCCOs* gene in rice (Priya et al., 2019). The results show that most *CCD* genes are expressed in seed and shoot tissues, implying that they are involved in important activities associated to these organs. Furthermore, the *CCDL* group expresses specifically in the seed and carpel, whereas the *NCED* group expresses specifically in the anther, carpel, and seed. This extensive analysis of gene expression data demonstrates the importance of the *OsCCOs* gene family in rice crop improvement. The discovery of their tissue-specific expression patterns opens the door to future breeding programs to produce new rice varieties with improved characteristics and attributes.

MicroRNAs (miRNAs) are crucial regulatory molecules in plants that play a significant role in almost all biological processes, including plant growth, development, and responses to biotic and abiotic stress (Ding et al., 2020). They are highly conserved and exhibit specific functions (Millar et al., 2019). The investigation showed that the *OsCCOs* has a variety of miRNAs with various roles, including by focusing on genes involved in nitrogen uptake and assimilation, Osa-miR169 controls how effectively the plant uses nitrogen (Peng et al., 2020). It is believed that Osa-miR5802 regulates rice grain yield and size (Kumar and Sanan-Mishra, 2022). By targeting genes associated with stress signaling and cellular homeostasis, Osa-miR1430 aids rice plants in surviving drought stress. Osa-miRN2366 is thought to be involved in controlling the growth and development of rice plants, while its exact role is still unclear (61). By focusing on genes involved in stress signaling and ion transport, Osa-miR1432 controls how rice plants react to salt stress (Zhai et al., 2020). Osa-miR2275 is an *OsCCD1* microRNA that has been connected to the production of siRNAs, small interfering RNAs that are unique to the inflorescence of rice (Fei et al., 2021). The possible implication from the observed results conveys that miRNAs could potentially govern the post-transcriptional control relating to Os*CCO* genes in rice progression.

This study’s outcomes provide fresh insights into the functional diversity and evolutionary dimensions of the CCO gene family in plants. These discoveries will serve as a valuable asset for future research, facilitating the functional examination and cloning of these genes. This comprehensive genome-wide identification and characterization, as undertaken in this study, will enable further investigations.

## 5 Conclusion

Sixteen Os*CCO* genes were discovered in *O.sativa* in this study. Based on structural analyses, the number of introns in *OsCCO* genes ranged from one to twelve. The presence of cis-regulatory elements related to light responsiveness, development-related response, developmental and hormone responsiveness, and certain abiotic stress in the promoter of *OsCCO* genes suggested their function in the abiotic stress of rice. *OsNCED6 and OsNCED10* genes identified by RNA-seq data analysis can potentially be utilized to develop salt stress-resistant rice varieties for enhanced crop yield under salt conditions. The *OsCCD7 and OsCCD8* genes are more active in inflorescences than in other organs, which suggests that they might play a role in grain development. However, additional research, including gene cloning and functional analysis, is required to confirm the significance of these genes in various physiological and biological processes. This in-silico analysis reveals the extensive genome-wide knowledge of rice *OsCCO* genes.

## Data availability statement

The original contributions presented in the study are included in the article/**Supplementary Material**. Further inquiries can be directed to the corresponding authors.

## Author contributions

MZ: Data curation, Formal Analysis, Writing – original draft. AS: Conceptualization, Data curation, Methodology, Writing – original draft. MS: Data curation, Formal Analysis, Software, Supervision, Writing – original draft. WA: Data curation, Investigation, Methodology, Writing – review & editing. SAli: Funding acquisition, Methodology, Resources, Writing – original draft. QA: Formal Analysis, Funding acquisition, Resources, Software, Writing – review & editing. SM: Writing – review & editing, Methodology, Resources. MS: Data curation, Funding acquisition, Investigation, Writing – review & editing. DA: Formal Analysis, Funding acquisition, Project administration, Supervision, Visualization, Writing – review & editing. SAla: Data curation, Funding acquisition, Methodology, Project administration, Resources, Writing – review & editing. IM: Writing – review & editing, Methodology, Formal Analysis, Software.
