# Supplementary material for: Genome-wide identification and in-silico expression analysis of carotenoid cleavage oxygenases gene family in Oryza sativa (rice) in response to abiotic stress
Source: Front Plant Sci. 2023 Oct 25;14:1269995. doi: 10.3389/fpls.2023.1269995 (PMC10634354; doi:10.3389/fpls.2023.1269995)
Supplement: Supplementary file 1 [file DataSheet_1.docx]

**Conserved Domain:**


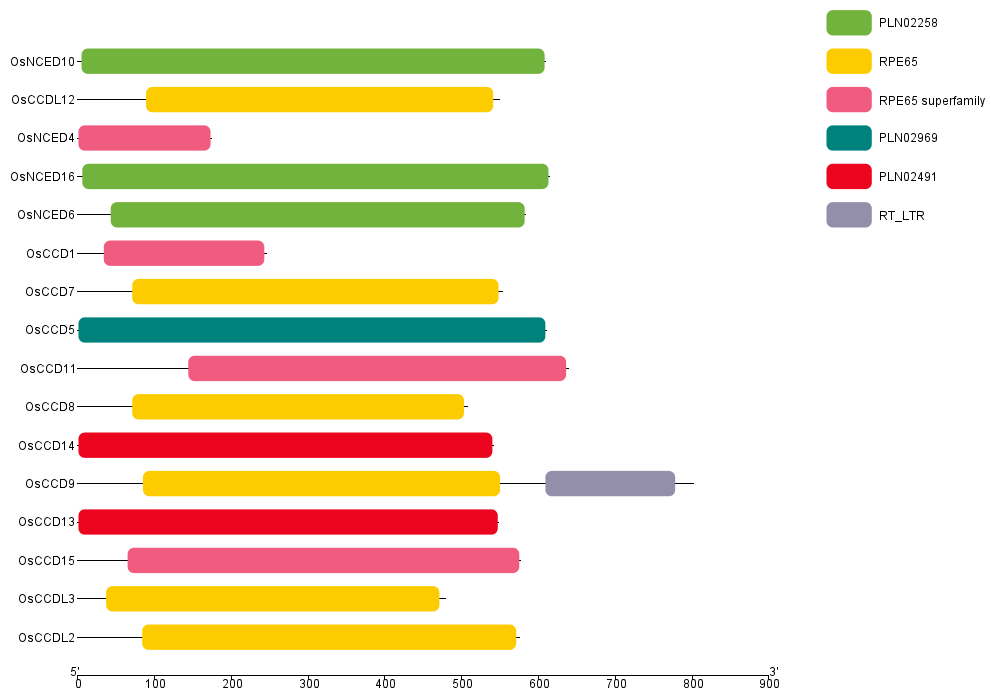


Figure 1S: Classification of domains within the *OsCCO* genes in rice.

**Conserved Motif Analysis:**


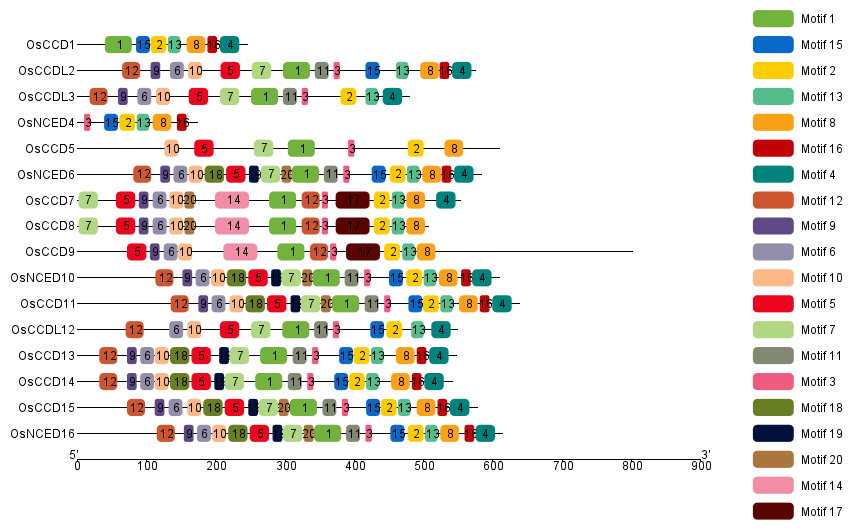


Figure 2S: Motif Profiles for OsCCO Genes
